# Extragalactic radio surveys in the pre-Square Kilometre Array era

**DOI:** 10.1098/rsos.170522

**Published:** 2017-07-19

**Authors:** Chris Simpson

**Affiliations:** Gemini Observatory, Northern Operations Center, 670 North A‘ōhōku Place, Hilo, HI 96720-2700, USA

**Keywords:** surveys, radio continuum: galaxies, galaxies: active

## Abstract

The era of the Square Kilometre Array is almost upon us, and pathfinder telescopes are already in operation. This brief review summarizes our current knowledge of extragalactic radio sources, accumulated through six decades of continuum surveys at the low-frequency end of the electromagnetic spectrum and the extensive complementary observations at other wavelengths necessary to gain this understanding. The relationships between radio survey data and surveys at other wavelengths are discussed. Some of the outstanding questions are identified and prospects over the next few years are outlined.

## 1. Introduction

Radio surveys provide a unique window to the distant Universe: the extremely strong evolution of the radio luminosity function (RLF) means that even shallow surveys contain distant objects. This was recognized in the early days of radio surveys, when the brightest extragalactic radio source, Cygnus A, was identified with a galaxy in a cluster at *z*=0.056 [1], making it one of the most distant objects known at the time. In comparison to galaxies in other distant clusters, the redshift of Cygnus A was very easily measured from its rich emission line spectrum. Although the invention of the photometric redshift method [2] allowed estimates of distances to normal galaxies too faint for spectroscopy, radio galaxies frequently displayed bright emission lines that provided easy-to-measure and accurate spectroscopic redshifts. When the redshift of 3C 295 was measured at *z*=0.4614 [3] this marked the beginning of a quarter of a century when a radio source marked the boundary of the known Universe.

A catalogued continuum radio source by itself only has position and intensity information and is of limited scientific value without an optical counterpart. With the error ellipses from early surveys typically several arcminutes in size, it was difficult to pinpoint the origin of the radio emission unless it was obviously associated with a bright galaxy or cluster of galaxies. Work therefore focused on the radio spectral energy distributions of sources, finding that most possessed a steep spectrum best explained by synchrotron emission, and analysis of the source counts showed strong evolution inconsistent with a Euclidean, Steady State Universe [4]. The construction of long-baseline interferometers allowed for more accurate positions and the ability to determine counterparts to a larger fraction of catalogued sources.

Of the many radio surveys undertaken during the late 1950s and 1960s, the 3CR survey [5,6], covering the northern sky to a bright 178 MHz flux density limit *S*_178_>9 Jy, has been perhaps the most enduring. Sources from surveys at fainter flux limits are either intrinsically less luminous (resulting in weaker emission lines and more difficult redshift determinations) or more distant (pushing the strongest emission lines beyond the limited range of optical spectrographs), and the shape of the source counts results in a rapid increase in source density on the sky with decreasing flux. The catalogue’s modest size also presented the possibility of high redshift completeness, although the final spectroscopic redshift in the more rigorously defined 3CRR catalogue [7] was not obtained until 1996 [8] and another was corrected a few years later [9]. And, despite its size, the strong evolution of the radio source population means that this catalogue contains a significant number of high-redshift objects—over one-fifth of its sources (37/173) have *z*>1, with one (3C 9) at *z*>2. This fraction of distant sources does not exist in a flux-limited catalogue at any other wavelength, which explains the reason for the use of radio surveys to search for distant objects.

Even in those early days, the number of catalogued radio sources far outstripped the ability to make robust optical identifications or perform spectroscopy of them. The specific follow-up of other surveys has depended on the scientific aims of the research groups involved. Some have sought to study the evolution of radio sources as a class and so require high redshift completeness, necessitating the study of selected areas of sky covered by the deeper radio surveys. Others have focused on identifying interesting objects (most notably very distant sources) and have developed ways to preferentially select such sources from the radio surveys, leaving the vast majority unstudied. More recently, groups have been able to use data from large public surveys to provide the necessary complementary data and, in the last few years, very deep radio observations have been undertaken in fields chosen because of their excellent multiwavelength data, specifically to benefit from these complementary imaging and spectroscopic observations.

The purpose of this review is not to comprehensively list every radio survey that has ever been undertaken. Instead it aims to summarize our current level of understanding regarding the multiple source populations that are detected in radio surveys while also providing historical context.

## 2. Powerful radio sources

Sub-arcminute imaging of bright extended extragalactic radio sources at low radio frequencies reveals them to broadly fall into two morphological classes. Those with the brightest regions near the centre of the source (edge-darkened) were named ‘Class I’ sources by Fanaroff & Riley, while those with the brightest regions near the furthest extent of the radio emission (edge-brightened) were called ‘Class II’ [10]. These have subsequently become known as FR I and FR II morphologies, after the authors of the study who found a strong correlation between morphological class and luminosity, with FR II sources being the most luminous. The boundary between the two classes is not sharply defined but there are roughly equal numbers of sources at a 178 MHz luminosity of *L*_178_∼10^26^ W Hz^−1^.

A second dichotomy among radio sources is also seen in their optical spectra. Sources either display rich emission-line spectra similar to the narrow-line spectra of Seyfert galaxies and quasars, or are almost entirely devoid of emission lines, possibly only displaying weak [Oii] *λ*3727 emission. Hine & Longair [11] named these ‘Class A’ and ‘Class B’ sources, respectively, and again radio luminosity is a key factor, with the most powerful sources belonging to Class A and the transition from Class B-dominated to Class A-dominated gradually taking place in the decade of luminosity above the Fanaroff–Riley break. This nomenclature never caught on and, when a quantitative classification method was first considered [12], the terms ‘high-excitation’ and ‘low-excitation’ were used to describe the spectra. ‘HE(R)G’ and ‘LE(R)G’, for high- and low-excitation (radio) galaxy, respectively, are now the most commonly used names in the literature. The similarity between the radio luminosities at which the transitions between Hine & Longair and Fanaroff & Riley classes occur is believed to be a coincidence but has led to frequent and erroneous use of the terms interchangeably. The FR class is also affected by extrinsic factors such as the circumgalactic density field [13,14], while the HERG/LERG classification depends on the availability of ionizing photons and hence the properties of the system only on parsec scales.

While early radio surveys were generally conducted at fairly low frequencies (approx. 100 MHz), samples were also constructed at higher frequencies, most notably the 2.7 GHz Parkes catalogue [15]. Surveys at different radio frequencies can produce significant differences in the samples selected. A powerful radio source consists of extended, steep-spectrum emission (typically *α*≈0.7, where *S*_*ν*_∝*ν*^−*α*^) and a jet-producing core with a much flatter spectrum (*α*≈0). The relative core-to-lobe flux ratio, *R*, therefore increases with increasing frequency. Although the intrinsic luminosity of the core is less than 1% of the total luminosity, even at a fairly high frequency like 5 GHz [16,17], its apparent luminosity can be dramatically increased by Doppler boosting, up to a factor of ∼2*γ*^4^, where *γ*∼5 is the Lorentz factor of the synchrotron-emitting particles in the jet [18,19]. A highly favourable orientation is required to boost the core emission by a sufficiently large factor to make its observed flux comparable to the extended emission, but the source counts are steep enough that bright high-frequency-selected samples have a significant fraction of sources with core-dominated morphologies. For example, the 178 MHz-selected 3CRR [7] and 2.7 GHz-selected Peacock & Wall [20] samples have similar source densities on the sky, but the fractions of sources whose morphology is dominated by an unresolved core at GHz frequencies are 11% (19/173) and 49% (83/168), respectively, while the fractions of flat spectrum (*α*<0.5) core-dominated sources are 4% (7/173) and 27% (46/168). Although there are only two sources in the 3CRR catalogue for which Doppler boosting of their cores has raised their total fluxes above the flux limit (3C 345 and 3C 454.3), the fraction is significantly higher in samples selected at GHz frequencies. High-frequency-selected samples are therefore not as clean as lower-frequency ones in providing the observed radio luminosity as a proxy for the intrinsic power of the central engine.

**Figure 1.**
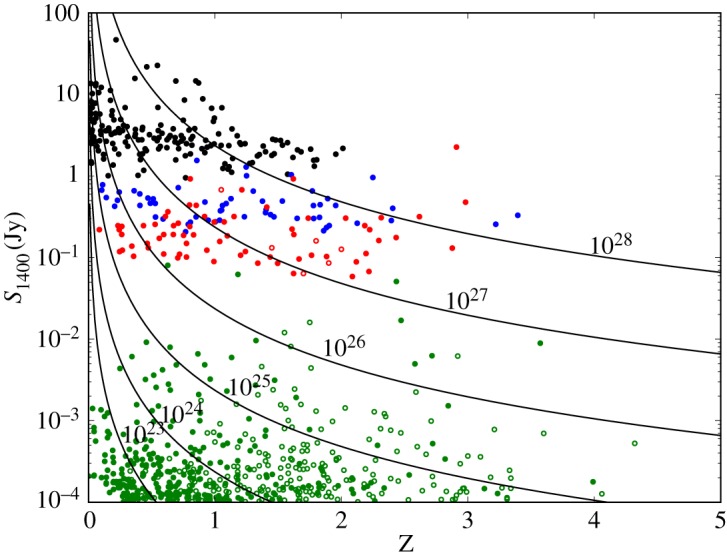
Observed 1400 MHz flux density against redshift for radio sources from four surveys: 3CRR [7] (black), 6CE [24] (blue), the 7C Redshift Survey [25] (red) and the Subaru/*XMM-Newton* Deep Survey [26] (green). While the SXDS survey has a flux limit at 1400 MHz, the other three surveys were selected and flux-limited at much lower frequencies. The existence of a correlation between radio spectral index and redshift results in the apparent 1400 MHz flux limits of these lower-frequency-selected samples decreasing with redshift, but it is impossible to determine from a single flux-limited sample whether the correlation is between spectral index and redshift or luminosity. Filled symbols indicate spectroscopic redshifts, while open symbols represent sources with redshifts estimated from broad-band photometry. Lines of constant 1400 MHz luminosity are shown in steps of one dex from $10^{23}\leq L_{1400}\leq 10^{28}\;W\;{\mathrm{Hz}}^{-1}$, as indicated (a spectral index *α*=0.7 and a flat *Λ*CDM cosmology with *H*_0_= 70 km s^−1^ Mpc^−1^ and Ω_m_=0.3 are assumed).

Even in very early studies with incomplete data there was evidence that the most luminous radio sources displayed a much stronger cosmic evolution than did lower-luminosity sources [21], and this result grew in strength as the data improved [7,22,23]. However, the steepness of the radio source counts at bright fluxes results in a very strong correlation between redshift and luminosity in any flux-limited sample since a large fraction of the sample is within a factor of two of the flux limit (three-quarters in the case of 3CRR, but only one-half for the much fainter SXDS sample; figure 1). In any single, reasonably sized flux-limited sample of a few hundred radio sources, it is therefore impossible to disentangle *redshift*-dependent effects from *luminosity*-dependent effects since the correlation between luminosity and redshift swamps all other relationships [27]. The use of multiple flux-limited surveys in a ‘wedding cake’ pattern allowed more complete sampling of the *L*–*z* plane and better observational constraints on the evolution of the radio luminosity function. Advances in our understanding of the unification of active galactic nuclei led to the positing of dual-population models, with HERGs and LERGs being treated separately and undergoing very different cosmic evolution [25].

## 3. Deep surveys and the faint radio source population

**Figure 2.**
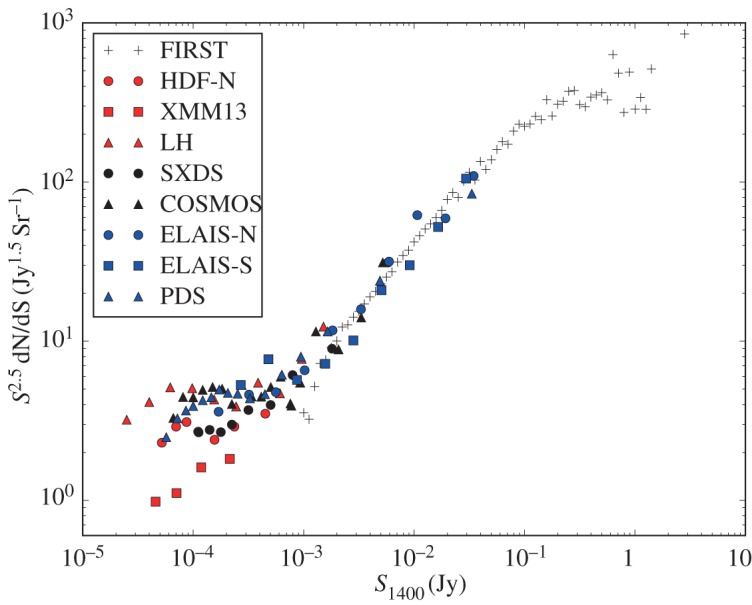
Euclidian-normalized source counts from selected 1.4 GHz radio surveys. To keep the figure clean, error bars are not plotted, but only points where the uncertainty, including sampling variance [29], is less than one-third of the measured counts are plotted. Plus symbols indicate counts from the Faint Images of the Radio Sky at Twenty Centimeters Survey (FIRST; [30]), while the other points have been colour-coded by survey area. Red points indicate counts from surveys covering approximately less than 1 deg^2^ of the Hubble Deep Field North [31], the *XMM-Newton* 13-h field [32] and the Lockman Hole [33]. Black points indicate surveys of approximately 1 deg^2^ from the Subaru/*XMM-Newton* Deep Survey [26] and the VLA-COSMOS survey [34]. Blue points indicate surveys of several square degrees, including the European Large Area *ISO* Survey Northern [35] and Southern [36] regions, and the Phoenix Deep Survey [37]. Note that the scatter at the faintest radio fluxes is much larger than predicted by cosmic variance [38].

The Leiden–Berkeley Deep Survey (LBDS) [28] consisted of nine pointings of the Westerbork Synthesis Radio Telescope (WSRT), in four regions of the sky with deep multicolour photographic plates, reaching sub-millijansky levels at a frequency of 1.4 GHz. Conducting the survey at this frequency provided not just the best sensitivity, but also resulted in more accurate positions, permitting reliable optical identifications. This became the frequency of choice for deep radio maps and the adjectives ‘microjansky’ or ‘sub-millijansky’ are normally assumed to refer to the flux densities of radio sources around this frequency, with 1 mJy being the flux density at which the source counts flatten (when plotted in a Euclidian-normalized manner, as in figure 2), indicating the presence of a new population. With the aid of Very Large Array (VLA) data, the LBDS probed well into this regime and the multicolour imaging revealed a change in the population at a flux density of a few mJy [39,40].

The counterparts to bright radio sources were dominated by red galaxies that followed the well-known Hubble relation for massive elliptical galaxies [41]. However, at the fainter flux densities where the source counts began to flatten, blue objects became an increasingly important contributor. These had lower radio luminosities than the red galaxies, and the more distant sources showed peculiar optical morphologies. Many objects, however, remained unidentified in the optical plates, limiting the general conclusions that could be drawn.

Attempts to further understand the nature of faint radio sources were hampered by the limitations of complementary data and available instrumentation. A notable effort [42,43] demonstrated the existence of a fairly heterogeneous mix of objects at $S_{1400}≲1\;\mathrm{mJy}$, although their reliance on optical counterparts from digitized photographic plates eliminated nearly 80% of their radio source sample from identification. They nevertheless concluded that spiral galaxies (predominantly star-forming objects, but including Seyferts) dominate the faint radio source counts. Linking these directly to the objects responsible for the *IRAS* 60 μm counts, they surmised that the strong evolution displayed is driven by galaxy interactions and mergers. A somewhat contrary conclusion was derived from a deeper (in both radio and optical limiting fluxes) radio survey of the Marano field, which concluded that the unidentified radio sources were probably distant elliptical galaxies [44].

Deeper optical imaging, including with the *Hubble Space Telescope*, provided much higher identification rates and detections at higher redshifts [45–47]. The morphological information granted by the *HST* images showed the hosts of the very faintest radio sources to typically be discs, often displaying signs of recent star formation. However, these studies were confined to very small areas of the sky, usually a few arcminutes across, and therefore most sources had flux densities s_1400_≪1 mJy. This is well below the source counts’ change in slope and fainter than the sources analysed in the earlier studies, leading to continued uncertainty as to the make-up of the population.

Another reason for the lack of progress towards a definitive understanding of the faint radio source population during the 1990s was sociological. For decades, the only spectroscopically confirmed high-redshift objects were radio sources and radio-quiet quasars, but the discovery of large numbers of distant galaxies via the Lyman break technique [48] changed this. Since the tight correlation between black hole and galaxy masses [49–51] had not yet been discovered, the black holes in active galaxies were viewed as a nuisance rather than a fundamental component of galaxy evolution. The study of extragalactic radio sources as a class quickly fell from favour and it became difficult to obtain telescope time for follow-up studies.

Progress was made by using existing or planned deep, wide-area extragalactic surveys to leverage new radio data. The prime-focus camera on Subaru Telescope, Suprime-Cam, was a key ingredient in these surveys as it provided an unprecedented combination of depth and area. From a radio perspective, these optical images could provide a high identification rate for statistically significant samples of radio sources at flux densities spanning the flattening in the source counts, and were therefore enormously helpful. Two fields provided the best ancillary data for studies of the faint radio source population: the Subaru/*XMM-Newton* Deep Survey (SXDS [52]) and the Cosmic Evolution Survey (COSMOS [53]). Covering more than one square degree each, they allowed identification of a sufficiently large sample of radio sources to quantify the composition of the microjansky population.

The radio survey of the SXDS [26] was undertaken with the VLA and comprised only 60 h of radio data, yet at the time it was the deepest degree-scale radio survey, and therefore the first to contain a meaningful number of radio sources with fluxes below the flattening in the source counts. The deep multicolour imaging provided reliable optical counterparts for all but a handful of sources and a simple visual inspection revealed a significant minority population of blue, star-like objects that were obviously optically luminous quasars. However, unlike the powerful radio-loud quasars found in shallow radio surveys whose radio luminosities are typically above the FR break, these objects were much less luminous, with ratios of radio to optical luminosity that put them clearly within the radio-quiet regime (see §6). The contribution to the radio source counts from this population had not previously been well determined; indeed, it had often been neglected, with models that did not include this population successfully fitting the observed counts [54]. On the other hand, estimates based on the observed X-ray source counts implied that it could be significant over a small flux range just below the flattening in the source counts, possibly dominant depending on the number of Compton-thick sources (which would contribute to the radio source counts but not the X-ray counts) [55]. The SXDS observations suggested that at least 20% of the sources just below the flattening of the source counts were luminous active galactic nuclei (AGN) that lacked powerful radio jets, and this estimate was subsequently supported by studies in the COSMOS field [56,57]. Although they do not dominate the source counts, this population follows the strong cosmic evolution traced by optically selected quasars and can therefore make a significant contribution to the RLF at redshifts *z*∼2.

## 4. Star-forming galaxies

Locally, radio emission is often observed to be associated with star formation, with synchrotron emission from electrons accelerated in supernova-driven shocks being the dominant component. The radio spectrum of a star-forming galaxy therefore has a similarly steep spectral index as an AGN, and both also flatten at higher frequencies due to the increased importance of a flat-spectrum component: the jet-producing core in an AGN, and thermal bremsstrahlung emission in a star-forming galaxy. As the cosmic star-formation rate density is known to increase rapidly with redshift [58], the radio sky should be full of star-forming galaxies.

As the pioneering work in the Hubble Deep Field showed [47], the dominant sources at the flux levels reached by the deepest surveys (s_1400_∼10 μJy) are star-forming galaxies spanning a range of redshifts. For galaxies at *z*>2 to be above the flux threshold of these surveys, they must possess star-formation rates (SFRs) of several hundred M_⊙_ yr^−1^, and this population therefore overlaps significantly with that of the submillimetre galaxies (SMGs). The tight correlation between the radio and far-infrared luminosities of star-forming galaxies [59] coupled with the vastly different *k*-corrections in the two wavelength regimes allows the ratio of radio-to-submillimetre flux densities to be used as a crude redshift indicator [60], with fainter radio sources (for a given submillimetre flux) likely to be more distant. As single-dish submillimetre detections have large astrometric uncertainties, the interferometric radio observations have been essential to localize the optical/near-infrared counterpart for additional analysis, including redshift determination. While some groups used the VLA to make complementary observations of regions that had already been surveyed at submillimetre wavelengths [61,62], others identified distant star-forming galaxies directly from their radio emission and optical faintness [63,64].

These radio detections were essential in permitting spectroscopic observations to determine redshifts and provide statistical information about the SMG population [65], although one thing that the high-resolution radio surveys failed to reveal was the high level of multiplicity among single-dish submillimetre sources (approx. 40%; [66]). The scatter in the far-infrared–radio correlation coupled with the modest signal-to-noise ratios of the radio detections makes it unlikely that more than one component would be detected if the single-dish detection was a blend of multiple sources. Nevertheless, the large primary beam of a radio antenna makes deep radio surveys an excellent way to produce large samples of star-forming galaxies at moderate-to-high redshift. Existing radio surveys are able to derive the luminosity function of star-forming galaxies out to *z*∼5 [67] (although the data do not probe below L* at *z*>2) and these objects will dominate the next generation of radio surveys.

## 5. Evolution of the radio luminosity function

Understanding the composition of radio surveys allows predictions for how the RLF will evolve, and what the radio source counts will be at currently unattainable flux levels, by using observations at other wavelengths. Such knowledge is important for designing surveys for future facilities like the SKA. Star formation in the Universe can be traced by infrared and ultraviolet continuum radiation and emission lines such as H*α* and [Oii], and accurate measurements exist to *z*>2 (a recent review is given in [68]). For radio-quiet AGN, the evolution of the optical quasar luminosity function has been well determined to *z*>5, and much telescope time has been awarded to X-ray surveys and spectroscopic follow-up to attempt to quantify the obscured AGN population. However, since X-ray emission can be affected by dust and/or gas absorption while radio emission is not, the uncertainty regarding the optically obscured and Compton-thick AGN fractions affects the predicted radio counts. The evolution of the most powerful (FR II) radio sources has been determined out to *z*∼3 from follow-up of radio surveys and, while uncertainty still remains over whether a ‘redshift cut-off’ exists, the effect of this uncertainty on the source counts is negligible.

**Figure 3.**
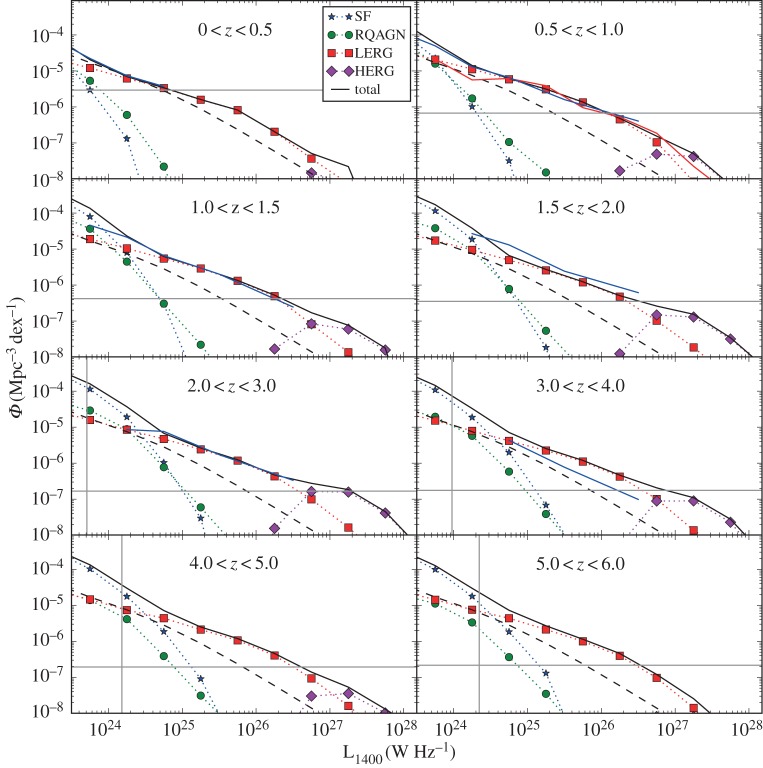
The 1.4 GHz radio luminosity function in different redshift ranges, as derived from the SKA Simulated Skies models [69]. The symbols denote different types of radio source as indicated by the key in the top-left panel, while the black solid line shows the overall luminosity function, and the black dashed line represents the parametrized redshift zero luminosity function from the 6-degree Field Galaxy Survey [70]. The red line in the 0.5<*z*<1.0 panel shows the observed radio luminosity function in this range [71], while the blue line in each of the first six panels shows the luminosity function in the Subaru/*XMM-Newton* Deep Field [72]. In each panel, the grey vertical line shows the luminosity of a source with s_1400_=10 μJy at the distant edge of the redshift bin (if absent, this limit is off the left-hand edge of the panel), while the grey horizontal line shows the space density corresponding to one source per square degree per luminosity bin.

The SKA Simulated Skies (S^3^ [69]) have used these observations to produce a model of the radio sky over a wide range of frequencies. At 1.4 GHz, the model extends down to 10 nJy, more than three orders of magnitude fainter than the deepest surveys currently in existence. At other frequencies, they surpass current data by even greater amounts. The prediction of this model for the evolution of the 1.4 GHz luminosity function is shown in figure 3, with some current observational measurements and limitations shown.

Significant progress has been made in measuring the evolution of the RLF by combining wide-area radio surveys with large spectroscopic datasets that provide redshifts and source classifications. In the very local Universe, the combination of the NRAO VLA Sky Survey (NVSS; [73]) with the 6-degree Field Galaxy Redshift Survey (6dFGRS) over 7000 deg^2^ has produced separate measurements of the RLF for AGNs and star-forming galaxies with *L*_1400_>10^20^ W Hz^−1^ [70]. More sensitive spectroscopic surveys have provided redshifts and classifications for sources out to *z*∼0.8 [74] which, although considered ‘modest’, represents a look-back time equal to half the age of the Universe. At *z*>1, however, it becomes increasingly difficult to determine redshifts and, particularly, classifications, since diagnostic features such as the [Oii] and [Oiii] emission lines move into the near-infrared.

Over much of the luminosity range shown the LERG population dominates, although the cosmic evolution of these sources has been the focus of fairly limited study because, unlike the other classes of source, they do not possess the strong emission lines that make redshift determination simple. Deep-field studies using photometric redshifts have suggested a decline in the space density of these sources beyond *z*∼1 [72,75], although work with higher spectroscopic completeness redshifts indicates this population experiences luminosity-dependent evolution, with the turnover taking place at *z*∼0.7 for sources below the characteristic luminosity of *L*_1400_≈10^26^ W Hz^−1^ [71,74]. These sources are interesting because they display the clearest evidence of AGN feedback as the radio-emitting plasma expands and creates ‘bubbles’ within the surrounding intergalactic medium [76,77]. It has been suggested that this form of AGN activity might be episodic in nature and, if so, the time-averaged heating rate has been shown to balance the cooling of gas from the hot halo, creating a self-regulating feedback loop that limits the stellar mass of galaxies [78,79].

Robust observational measurements of the RLF at high redshifts and modest luminosities are limited by the availability of complementary data at other wavelengths and, in particular, spectroscopy. Even when a spectrum is available, it often fails to provide a redshift, let alone a classification. Unlike the powerful FR II HERGs that dominate the bright radio surveys at these redshifts, the microjansky sources usually have weaker emission lines, and fewer of them, because star-formation processes do not produce the rich spectrum of an AGN, and the emission-line producing region is often completely obscured by dust [80]. Photometric redshifts are therefore an essential part of any study, but all studies have a few per cent of ‘catastrophic outliers’ among the objects with spectroscopic redshifts and, of course, these are not an unbiased subset, so the true fraction of such sources is unknown. While increasing the sample size and/or using the full posterior *P*(*z*) distribution can mitigate the effect of random photometric redshift errors on the determination of luminosity functions, these do not alleviate the errors introduced by outliers. Apart from the primary effects of assigning an incorrect redshift, and therefore luminosity, to a particular source, there can be secondary effects. For example, the ratio of rest-frame 24 μm to radio luminosity, *q*_24_, is often used to discriminate between radio-loud and radio-quiet sources [81], but the 9.7 μm silicate absorption feature passes through the 24 μm filter bandpass at *z*∼1.5, making the *k*-correction very sensitive to the adopted redshift and possibly causing the misclassification of sources.

A final, yet important, concern regarding a comprehensive analysis of the faint radio source counts arises from a lack of uniformity in the measurement of source fluxes. The scatter observed between the different measurements of the source counts in figure 2 is much larger than can be attributed to cosmic variance [29,38]. Most source flux densities are determined (at least as a first estimate) from an elliptical Gaussian fit, where there exist correlations between the fitted parameters. For a genuinely unresolved source, these fits overestimate the true flux density if the best-fitting source size is larger than the beam, and underestimate it if the source is smaller than the beam [82]. However, some authors ignore the fit when it produces a source size smaller than the beam (which is obviously unrealistic) and adopt the peak value as the flux density, but this overestimates the true flux density. Consequently, the fluxes of *all* unresolved sources are overestimated, except in the very unlikely case that the fitted source size is exactly the beam size. With the source counts being so steep below 1 mJy, systematic overestimates of source fluxes are amplified when used to derive the source counts: since the counts are roughly flat when plotted in the manner of figure 2, a 10% overestimate of the source fluxes will result in a 27% overestimate of the source counts, and even larger flux errors are possible (see fig. 4 of ref. [26]). Furthermore, resolved sources can be missed in a catalogue if their peak flux densities are below the detection threshold, and correcting for this incompleteness requires knowledge of the true source size distribution, which remains rather uncertain [83,84]. Recovering integrated flux densities from a tapered lower-resolution map is impractical in the deepest surveys due to confusion, and it is vital that agreement is reached on how to measure reliable source fluxes from interferometric data, or the scientific usefulness of these data will be severely compromised.

## 6. The radio-loud/radio-quiet distinction

One of the major questions in extragalactic astronomy is the origin of radio-loudness. Although the first quasars were discovered because of their powerful radio emission, most optically selected quasars were too faint to be detected at radio wavelengths. Once the VLA enabled sensitive observations of large samples of optically selected quasars, a dichotomy in their radio properties appeared. Defining radio-loudness, *R* as the ratio of radio to optical luminosities (typically *B*-band and 5 GHz), there appeared to be a dearth of sources with *R*≈1–10 [85,86].^1^ Sources with large values of *R* were determined to be an orientationally biased subset of powerful FR II radio galaxies, with their true radio structures often obscured by the powerful Doppler boosting of the core.

Radio luminosity is easy to measure but is known to be only a small fraction of the energy needed to power the jets in these powerful sources. Two methods for estimating the kinetic power of the large-scale radio jets have been widely used in the literature. One uses the minimum energy condition to estimate the energy stored in the particles and magnetic field [87], and then divides this by the estimated age of the radio source [88]. A second, more recently developed, method is applicable in cases where the radio source has excavated bubbles in the surrounding X-ray-emitting plasma. Here, the *p* *dV* work done in excavating the bubbles is estimated and, again, is divided by the estimated age of the source [89,90]. Given the observed scatter and theoretical uncertainties in these relationships, the precise normalization of the relationship between radio luminosity and kinetic jet power is uncertain by a factor of a few but, in general, results support a relationship $Q\propto L_{\nu }^{\beta }$, where *β*≈0.8 [91]. Estimates for powerful radio-loud sources suggest that the efficiency for converting kinetic power, *Q*, to radio luminosity, *νL*_*ν*_, is of the order of 1%, assuming that the jets contain only light particles [92], and this power is comparable to the total luminosity in ionizing photons [93].

The ability to use radio luminosity as a proxy for the accretion power in powerful radio sources, albeit with significant normalization uncertainties, is highly beneficial since the radio photons are unaffected by intervening dust or gas, but it remains unclear whether there is a similar relationship for radio-quiet objects. In radio-loud sources, the radio emission unequivocally arises from synchrotron-emitting plasma that is transported by large-scale jets. The radio emission in radio-quiet objects is much smaller in extent and approximately three orders of magnitude lower in luminosity, when normalized by an accretion-rate-dependent quantity such as optical luminosity (for quasars) or narrow-line luminosity. This naively suggests that radio-quiet sources have an energy input into synchrotron-emitting particles approximately 1000 times lower, but that assumes the physics are the same, and it is unclear whether the radio emission in these objects arises from plasma accelerated in jets. There have been two recent suggestions for non-jet origins of the radio emission in radio-quiet objects. Similarities between the shapes of the RLFs for nearby galaxies and low-redshift quasars were used to suggest that the radio emission in quasars might arise from star formation [94]. Such objects would only need modest star formation rates of around 20 *M*_⊙_ yr^−1^ but distant luminous quasars would require rates of hundreds of *M*_⊙_ yr^−1^ [95]. With mid-infrared observations apparently ruling this out, it has instead been suggested that the radio emission in radio-quiet sources may arise from wind-driven shocks, which would be expected to have a very low radiative efficiency by analogy with supernova-driven shocks [96]. While radio-quiet quasars display a range of morphologies, some display very clear jet-like features and appear to simply be scaled-down versions of their radio-loud cousins [97,98]. In nearby Seyfert galaxies, where the physical resolution is better, linear structures are seen in three-quarters of objects whose radio emission is resolved [99]. There is also a tendency for linear structures to be more prevalent in more radio-luminous objects, which could arise either as a consequence of the correlation between linear size and radio luminosity coupled with limited angular resolution, or simply because lower-luminosity jet-like features can be more easily swamped by the radio emission from the star formation that is often associated with AGN activity.

Perhaps the most compelling evidence for jets in radio-quiet quasars comes from VLBI observations that reveal extremely high brightness temperature cores [100,101], while the abundance and properties of optically selected flat-spectrum quasars are most readily explained if the radio emission arises from Doppler boosting, requiring the jets to be relativistic [102,103]. The quasar E1821+643 is a particularly interesting source because it has such a high optical luminosity (*M*_*B*_≈−27) that, despite its radio-quiet nature, its radio luminosity is similar to that of nearby FR I radio galaxies, and it displays a similar radio morphology [104,105]. This suggests that perhaps radio-quiet active galaxies simply supply a much smaller fraction of their accretion energy to producing jets, which are otherwise identical to those of radio-loud objects. Alternatively, the particular make-up of the jets might differ, with ‘light’ electron–positron jets being launched in radio-loud sources and ‘heavy’ electron–proton jets launched in radio-quiet ones. The kinetic jet powers of both types of source would therefore be similar, but approximately 99.9% of the kinetic energy in radio-quiet objects would be used to accelerate protons that emit negligible synchrotron emission.

Could the difference in the synchrotron luminosities of radio-loud and radio-quiet objects be driven by a difference in the properties of the supermassive black holes that power them? It is known that radio-loud objects are limited to the sources with the highest accretion rates, as identified by [Oiii] emission [97], and the highest black hole masses [106,107], but these extreme objects are not *exclusively* radio-loud. It has also been known for many years that the hosts of powerful radio-loud objects are exclusively elliptical galaxies and these facts have been brought together by a number of authors to suggest that the energy source for powerful radio jets may be the rotational angular momentum of the black hole [108–111]. If the primary route for creating rapidly spinning black holes is via the merger of two similar-mass black holes (presumably hosted in two similar-mass galaxies) where orbital angular momentum is converted to rotational angular momentum during coalescence, then it follows that such objects will reside in ellipticals (the product of major galaxy mergers) and will typically have higher masses than the black holes that power radio-quiet sources.

While this is a qualitatively attractive model, it suffers from the lack of a quantitative formalism for the energetics of jet production. In addition, indirect measurements of black hole spin in nearby Seyfert galaxies are high despite the radio-quiet nature of these objects [112–114], although doubt has been cast on the validity of these results [115]. An alternative idea suggests the radio-loud and radio-quiet states are a form of duty cycle analogous to those seen in X-ray binaries [116–118]. An apparent prediction of this scenario is that AGN should be able to shut off and then restart in a different mode, in particular to transition from radio-loud to radio-quiet. While there are known to be sources that stop and restart their activity while remaining radio-loud (the so-called ‘double–double radio galaxies’), no sources are known where a radio-quiet AGN is surrounded by relic lobes from a prior radio-loud episode of activity.

## 7. Distant radio sources

There already exist a number of excellent reviews of high-redshift radio galaxies (HzRGs) [119,120]. Known examples of these objects are exclusively luminous high-excitation FR II sources whose bright emission lines (including Ly*α*) allow for easy redshift measurements. Apart from the general desire of astronomers to hold the record for discovering the ‘most…’ something, there are compelling scientific reasons for searching for HzRGs: the evolution of the radio galaxy population at early cosmic times will reveal details about the formation mechanism of these rare objects; being powered by the most massive supermassive black holes, they are likely to form at density peaks and provide signposts to galaxy protoclusters; and sufficiently distant objects (*z*>6) can act as background radiation sources against which to study reionization via the 21 cm forest.

Given the extreme rarity of such objects among the general radio source population, various criteria have been developed to improve the efficiency of HzRG searches by removing objects likely to be in the foreground. Typically sources larger than approximately 15′′ are excluded, due to an observed anti-correlation between redshift and angular size [121] and, since distant radio galaxies are also found to have steeper spectral indices [122], a spectral-index cut is also applied. Following up only sources with *α*>1 enabled the identification of the first *z*>2 radio galaxies [123,124] and even more aggressive spectral index cuts produce a higher fraction of the highest redshift sources (*z*>3), with the extreme criterion of *α*>1.3 enabling the discovery of the most distant radio galaxy identified to date, TN J0924–2201 at *z*=5.19 [125,126]. Of course, such aggressively selected samples suffer from hard-to-quantify incompleteness and cannot be reliably used to estimate the space density of HzRGs [127].

By modelling the evolution of powerful radio sources, the reason for these correlations with redshift was traced to dramatically increased inverse Compton losses to the cosmic microwave background (the energy density of the CMB increases ∝(1+*z*)^4^) [27,128,129]. As more energetic electrons (which emit their synchrotron radiation at higher frequencies) lose energy to inverse Compton scattering more rapidly, this results in the radio spectrum becoming steeper and more curved, as well as decreasing in luminosity. As a single observing frequency samples increasing rest-frame frequencies for higher redshift objects, the HzRG fraction will be largest in low-frequency selected catalogues.

Even with these selection criteria, a sample optimized for finding HzRGs based solely on radio properties will still contain many lower-redshift interlopers. Only optical or near-infrared imaging can reliably exclude these objects, as true HzRGs will be very faint at these wavelengths. *K*-band imaging is most frequently used because radio galaxies have a tight locus in the near-infrared Hubble diagram and the so-called ‘*K*–*z* relation’ [130] can be used to give a very crude redshift estimate, often providing support for the identification of a single emission line as Ly*α* rather than [Oii] at a much lower redshift.

The Hubble diagram for radio galaxies was originally developed to measure the Hubble constant, under the assumption that the sources were all first-ranked ellipticals and could be used as standard candles [41,131]. Historically, therefore, it was parametrized as m(*z*), with the magnitude measured in an aperture of fixed linear size of 63.9 kpc, corresponding to approximately 8′′ at high redshift. Given the faintness of distant radio galaxies, the unacceptable loss of signal-to-noise ratio incurred from the use of such a large aperture requires authors to make measurements in smaller apertures and correct these to the standard aperture using a curve of growth. As distant radio galaxies clearly do not follow this smooth surface brightness profile, the results are dependent on the actual aperture used, as well as the adopted curve of growth and cosmology. Progress has been slow in moving towards a more practical Hubble diagram, using magnitudes in a fixed angular aperture [132], as well as fitting the redshift as a function of magnitude [133], which is more appropriate as the diagram is now used to estimate redshifts, with the observed magnitude being the independent variable.

Obtaining the deep imaging needed to exclude low-redshift sources is the most expensive step (in terms of telescope time) in the identification process. With typically an hour of 8 m telescope time required per source, very severe size and spectral index cuts are needed to filter a sample to provide high efficiency, at the expense of completeness. Rather than pursue bespoke imaging for filtered samples of radio sources, however, it is possible to use existing deep near-infrared imaging with less severe radio selection, allowing a reliable measurement of the space density of radio galaxies in the early Universe. Cross-correlating the *Spitzer Space Telescope*’s SWIRE survey [134] with the FIRST [30] survey produced a sample of infrared-faint radio galaxies over 24 deg^2^ with counterparts ready for spectroscopy. Although the 1.4 GHz frequency of the FIRST survey is not optimal for finding HzRGs, three new *z*>4 radio galaxies were identified [135], including the second most distant radio galaxy known [136]. This source, FIRST J163912.11 + 405236.5 at *z*=4.88, has a spectral index between 325 MHz and 1.4 GHz of 0.75, which would have excluded it from any sample filtered by spectral index. A larger sample of objects selected with similar criteria further demonstrated these objects do not typically have ultra-steep radio spectra [137], although the very faintest infrared sources typically do have *α*>1.0, suggesting they may lie at the very highest redshifts [138].

Although the record for the most distant radio source has stood for nearly two decades, models predict approximately 200 radio galaxies with *z*>6 and s_150_>10 mJy over the sky. Such sources can provide a unique insight into the Epoch of Reionization (EoR) by observing the 21 cm forest in absorption [139]. This is analogous to the use of the Ly*α* forest in absorption along the lines of sight to optically bright quasars [140] but is much less limited. Whereas a neutral fraction as low as around 0.01% leads to an opaque Gunn–Peterson trough within which even the deepest spectroscopic observations cannot detect any flux [141], the 21 cm hyperfine transition has a cross-section around 10^7^ times smaller than Ly*α*, and even predominantly neutral gas transmits radiation, allowing the clumpiness of the intergalactic medium to be studied. Optical/near-infrared searches for high-redshift quasars continue to discover new sources at *z*>6 but these are all radio-quiet and therefore too faint for 21 cm forest studies. It is to be hoped that, as the number of such sources increases approximately towards 100, one or more is found to be radio-loud simply by chance.

At less extreme redshifts, the apparent tendency of radio galaxies to live in dense environments and hence act as signposts to protoclusters provides another reason to search for them. The first observational clues that at least some HzRGs lived in dense environments came from the large rotation measures observed [142]. Deep imaging often reveals an excess of companion sources, identified via photometric redshifts, narrow-band imaging for Ly*α* or H*α* emission, or spectroscopy. While a significant number of HzRGs are known to reside in protoclusters, including TN J0924–2201 [143,144] and the well-studied ‘Spiderweb’ galaxy PKS 1138–262 [145,146], there is no measurement of the fraction of HzRGs that live in such environments from an unbiased sample. Furthermore, since the lifetime of the radio source (approx. 10^8^ yr) is much less than the cluster collapse time (a few Gyr), the epoch of radio source activity may highlight a specific period in the formation of the cluster. While these distant protoclusters therefore provide interesting laboratories in the early Universe, one must be cautious of possible biases that might preclude generalizing any results.

## 8. The future

Three new radio telescopes are in the process of conducting major continuum surveys with equivalent depths s_1400_≪1 mJy over large regions of the sky. These surveys will be dominated by star-forming galaxies and a key requirement in extracting the maximum scientific return will be identifying and separating these objects from other classes such as radio-quiet AGN. Although high-resolution radio observations (approx. 0.1′′, requiring 500 km baselines at 1.4 GHz) can reveal the presence of an AGN that eludes detection at all other wavelengths [147], the surveys have resolutions of a few arcseconds, preventing the use of radio morphology as a discriminant, and the radio spectra of both classes of source are similar. Spectroscopy will be useful in assigning objects to the ‘mainly star-forming’ or ‘mainly AGN’ bins but this is still prone to bias from dust obscuration. Given the vast sizes of the radio source samples created by the pathfinders, follow-up is likely to be either statistical, with subsamples used as training sets for machine learning, or focused on unusual objects that lie outside the normal parameter space.

The Australian SKA Pathfinder, ASKAP,^2^ consists of thirty-six 12 m antennas equipped with phased array feeds that dramatically increases its survey speed. Among several Survey Science Projects, the Evolutionary Map of the Universe survey (EMU [148]) is an ‘all-sky’ (3*π* steradians) survey at 1.3 GHz with 10′′ resolution and 10 μJy RMS. The reliability of identifying the optical/infrared counterparts to radio sources does not suffer significantly at this resolution, but the depth of the complementary data has a major effect [149]. This will be the main limitation in trying to extract the maximum from all-sky surveys and, while the Large Synoptic Survey Telescope (LSST) will ultimately survey the entire southern sky to *r*∼27.5, similar to the SXDS optical data, these observations will not be completed until the 2030s.

The African SKA precursor telescope, MeerKAT,^3^ will comprise sixty-four 13.5 m antennas. These are fitted with single-pixel detectors, making the telescope better suited to deep surveys over small areas. Most relevant to this article is the MIGHTEE survey, which will cover 20 deg^2^ at 1.4 GHz to a depth of 1 μJy RMS in fields where there is the best complementary data, including the LSST deep-drilling fields and those with near-infrared imaging from the VIDEO survey [150], to aid with the cross-identifications.

The low-frequency array (LOFAR^4^) is already conducting surveys between 20 and 200 MHz, with its most sensitive observations around 150 MHz. It is undertaking several nested surveys at multiple frequencies, from a shallow ‘all-sky’ survey to a deep survey covering a few individual pointings centred on fields with excellent complementary data. It also has a dedicated spectroscopic follow-up programme, the WEAVE/LOFAR survey [151], which will produce approximately one million spectroscopic redshifts for radio sources selected at 150 MHz. The low-frequency selection favours the identification of the highest redshift sources, and the widest tier of the WEAVE/LOFAR survey is expected to find tens of radio galaxies at *z*>6, producing multiple lines of sight to probe the structure of the intergalactic medium during the Epoch of Reionization using the 21 cm forest.

In addition to these new facilities, the Karl G. Jansky Very Large Array is undertaking the VLA Sky Survey (VLASS^5^), a 3 GHz survey covering 82% of the celestial sphere in two epochs with a resolution of 3′′ and a depth of 80 μJy RMS. These four projects together provide complementary new windows on the radio sky from their sky area, depth and/or observing frequency, and will help to define the optimal SKA surveys as well as answering scientific questions themselves.

Over the past decade, there has already been a gradual shift in the use of radio data, as its synergy with imaging at other wavelengths has been exploited. As we approach and enter the era of the SKA, the properties of the radio sky should become essential information for all extragalactic astronomers. The density of radio sources on the sky with s_1400_>1 μJy is comparable to the number of galaxies with *V* <24 so radio fluxes will be available for the majority of objects detected in optical/near-infrared surveys and represent important photometric data, providing a dust-independent measurement of the star-formation rate, or indicating the presence of an AGN. Modelling the radio sky in simulations and semi-analytic models should also be a goal, but this requires an understanding of the physical processes that trigger the radio emission from galaxies, and this is still highly uncertain except for star-formation. As supermassive black holes are now included in simulations and semi-analytic models, there is a possibility that, as these models mature, they could help to reveal the cause of the radio-loud/radio-quiet dichotomy in AGN.

As much of a challenge as the raw data processing requirements of these new instruments will be learning how to use the vast array of information efficiently to answer questions, some of which have not even been posed yet. Radio astronomy has often been seen as an obscure and separate discipline but the next few years represent a key period in integrating the subject into the mainstream. The Square Kilometre Array will be a truly global telescope and its data should be owned by everybody.

## Footnotes

1 Some authors have tried to use this ratio as a diagnostic for non-quasar sources but the optical emission of these objects arises from the stellar population, whose luminosity does not generally correlate with accretion power (although for Eddington-limited sources, the two are connected via the central black hole mass).

2 http://www.atnf.csiro.au/projects/askap

3 http://www.ska.ac.za

4 http://www.lofar.org

5 http://science.nrao.edu/science/surveys/vlass

## Data accessibility

This article has no additional data.
